# Preparation of Magnetic Polymers for the Elimination of 3-Isobutyl-2-methoxypyrazine from Wine

**DOI:** 10.3390/molecules23051140

**Published:** 2018-05-10

**Authors:** Chen Liang, David W. Jeffery, Dennis K. Taylor

**Affiliations:** ARC Training Centre for Innovative Wine Production and School of Agriculture, Food and Wine, The University of Adelaide, Waite Campus, PMB 1, Glen Osmond SA 5064, Australia; c.liang@adelaide.edu.au (C.L.); dennis.taylor@adelaide.edu.au (D.K.T.)

**Keywords:** polymer synthesis, wine flavour, adsorption isotherm, adsorption kinetics, reusability

## Abstract

3-Isobutyl-2-methoxypyrazine (IBMP), the most prevalent grape-derived methoxypyrazine, can contribute green bell pepper, vegetative and herbaceous aromas and flavours to wines. At elevated concentrations, this potent odorant may mask desirable fruity and floral aromas in wine and may be considered as a fault. A new remediation method for wines with elevated IBMP levels has been trialled using magnetic polymers, prepared in the same way as ordinary polymers but with the incorporation of iron oxide nanoparticles as magnetic substrates. Characterisation by Fourier transform-infrared (FTIR) spectroscopy and scanning electron microscopy (SEM) showed no difference between thermally synthesised and microwave synthesised polymers. Magnetic polymers were found to have removed over 40% of the IBMP present in spiked model wine and white wine within ten minutes. The addition of magnetic nanoparticles and microwave-induced polymerisation did not affect the adsorption properties of the polymer in model wine and the polymer could be regenerated at least five times. Both Langmuir and Freundlich isotherms were found to fit the data for both types of polymer. However, attempts to produce imprinted polymers were not achieved, as they were found not to be differentiated from non-imprinted counterparts via adsorption tests.

## 1. Introduction

Alkylmethoxypyrazines (MPs) are aroma volatiles noted for their potency and ability to impart sensory characters of green bell pepper, grass, and vegetables to wine [1]. Three grape-derived MPs have been uncovered in recent decades—3-isobutyl-2-methoxypyrazine (IBMP), 3-isopropyl-2-methoxypyrazine (IPMP) and 3-*sec*-butyl-2-methoxypyrazine (SBMP) [1]—that are mainly located in grape stems, followed by skins and seeds [2]. Another source of MPs in wines originates from the contamination of grapes by Coccinellidae beetles, leading to a wine fault known as ‘ladybug taint’ (LBT) [3,4]. More recently, 2,5-dimethyl-3-methoxypyrazine (DMMP) was identified as another MP compound released by Coccinellidae that contributes to LBT odour [5]. MPs can contribute to the so-called varietal flavours of certain grape varieties including Cabernet Sauvignon, Cabernet Franc, Sauvignon Blanc, and Carmenere [6]; however, high levels (≥20 ng/L) can be overpowering and cause undesirable ‘green’ and ‘unripe’ characters [7] due to the extremely low sensory thresholds of MPs. The detection and recognition thresholds of IBMP determined in red wine are 10 ng/L and 15 ng/L, respectively [8]. The quantity of IBMP is constantly found to be higher than IPMP and SBMP in grapes and wines, and ranges in wines from below 2 ng/L to around 50 ng/L [9]. Consequently, the level of IBMP may be utilised as an indicator of the overall green character potential of grapes and wines.

Grape IBMP concentrations can be affected by grape maturity, sunlight exposure, water status, temperature, vine vigour, and yield [10,11,12,13]. It has been confirmed that greater light exposure for the berries before veraison can decrease IBMP accumulation [12], but changes during ripening have not been explained entirely. The decrease in IBMP concentration during berry maturation may be mainly driven by dilution due to an increase in berry weight [14], with no clear degradation pathway of IBMP thus far being elucidated.

Since IBMP is relatively stable during fermentation and ageing [15,16], remedial methods are necessary when there are highly elevated IBMP levels, for example, in grapes from cool climate regions or when grapes are picked early to make lower alcohol wines. Several pre- or post-fermentation treatments to remove excessive MPs from juice or wine have been investigated. Must clarification is reported to remove 50% of IBMP in grape juice [2], but this is not suitable for skin-fermented wines. Fining agents such as bentonite and activated charcoal have little effect on the concentration of MPs in wine and lack selectivity [17]. In recent years, several kinds of polymers have been used to remediate juice or wine with excessive MPs [18,19], and silicone and polylactic acid polymers have been reported to be able to remove grape-derived MPs in wine while causing little change in most non-targeted volatile aroma compounds and colour parameters [20]. Addressing the issue of selectivity, molecularly imprinted polymers (MIPs) have been utilised for the extraction of MPs from wine [21,22,23].

MIPs offer a promising alternative to traditional solid-phase sorbents by possessing complementary cavities for target molecules such as IBMP. By association and then disassociation of a template (target molecule or target analogue) during synthesis, this group of polymers gains “memories” of the target molecule and can therefore bind specifically with that compound [24]. Furthermore, MIPs can be made into magnetic forms, termed magnetic molecularly imprinted polymers (MMIPs), through the attachment of magnetic substrates [25]. The major advantage of using MMIPs is they can be directly separated by an external magnetic field instead of by filtration. Magnetic polymers have been applied to extract inorganic food components such as heavy metals and organic components including veterinary drugs, pesticides, and hormones [26]. Magnetic particles coated with MIPs have been used to extract resveratrol from red wine [25], Sudan dyes from chili powder [27], bisphenol A in milk [28], and vanillin in food samples [29].

In this study, attempts were made to produce synthetic MMIPs for IBMP extraction from wine, with a comparison to magnetic non-imprinted polymers (MNIPs), and to non-magnetic counterparts for what appears to be the first time. In addition, microwave-induced polymerisation versus conventional thermal synthesis was evaluated. Physical characterisation and adsorption analysis were carried out to evaluate the different polymers.

## 2. Results and Discussion

### 2.1. Preparation of Polymers

Polymers were prepared as outlined in Section 3.2. In an attempt to produce molecularly imprinted polymers (referred to as MMIPs throughout to differentiate from polymers produced without the use of a template), 2-methoxypyrazine was employed as a template to overcome the ‘template bleeding’ problem that occurs when IBMP has been utilised as the template in previous trials [23]. That said, even with continuous multiple solvent extraction, an equilibrium may be reached where there is always residual template left in the polymer, which would leak into the solutions during adsorption tests [30]. In other cases, µg or ng levels of residual template would be an acceptable level of bleeding for the analysis of compounds in the mg range. However, in the case of IBMP and the ultra-trace levels present in grapes and wines, ng levels of bleeding of IBMP would be unacceptable and greatly affect adsorption tests conducted within a practical concentration range [31]. Thus, choosing an analogue to IBMP as a template was deemed to be the better way to solve the template bleeding problem [32,33], as ultra-trace levels of 2-methoxypyrazine leaching would not interfere with the adsorption analysis. From a practical consideration, it should be a food-grade chemical with a much higher sensory threshold than IBMP so it would not be detected at trace levels [34]. Microwave synthesis was trialled due to its noticeable time-saving and consistent performance [35]. The synthetic process went smoothly for both microwave (MW) and conventional methods and the finished products were similar in appearance to bulk polymerisation products.

### 2.2. Characterisation of Polymers

**Figure 1:**
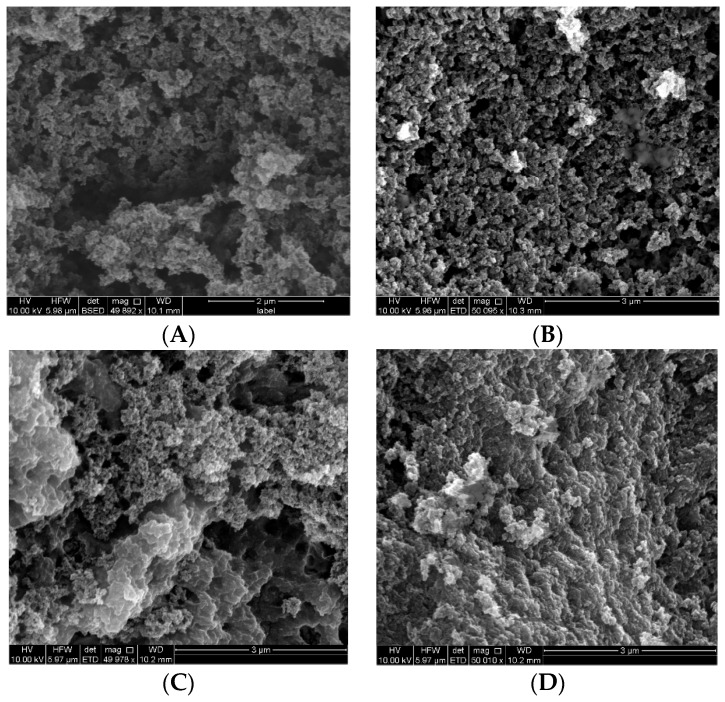
Scanning electron microscopy (SEM) images of polymers prepared by conventional thermal synthesis for (**A**) molecularly imprinted polymer (MIP) and non-imprinted polymer (NIP) (**B**), and by microwave (MW) synthesis for (**C**) MW MIP and (**D**) MW NIP.

**Figure 2:**
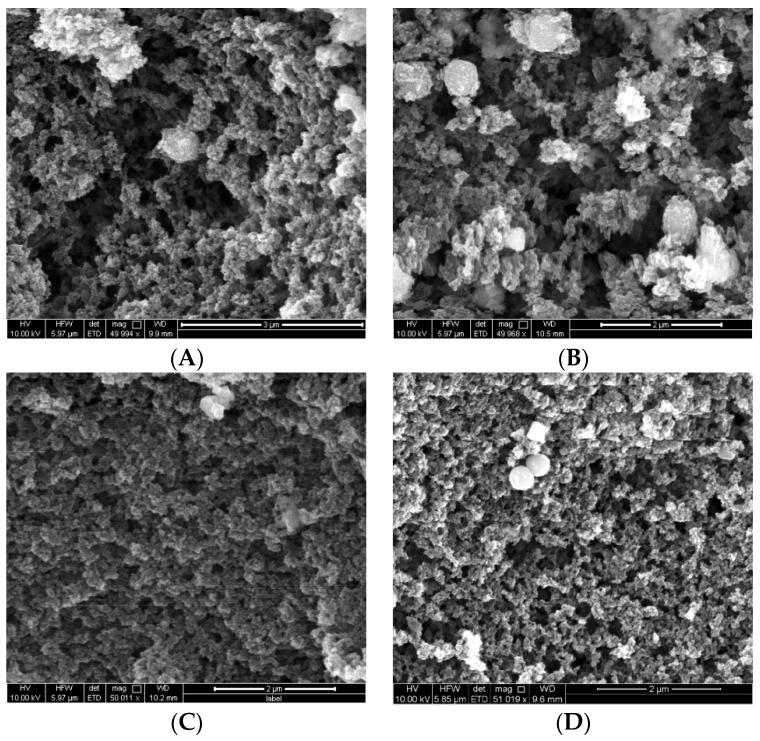
SEM images of magnetic polymers prepared by conventional thermal synthesis for (**A**) magnetic molecularly imprinted polymer MMIP and (**B**) magnetic non-imprinted polymer (MNIP), and by MW synthesis for (**C**) MW MMIP and (**D**) MW MNIP.

Figure 1 shows scanning electron microscopy (SEM) images of the various polymers produced by conventional and MW synthesis. The polymers were deemed to be micro- to meso-porous (<2 nm to 2–50 nm) and no backbone structural difference regarding compactness was found between the imprinted and non-imprinted polymers, nor with the MW synthesised polymers. In comparison to the regular polymers, images of the magnetic polymers implied the presence of metal (bright spheres, Figure 2) due to the incorporation of iron oxide nanoparticles (using commercial iron (II,III) oxide nanoparticles, which may or may not be purely magnetite in the products, so Fe_x_O_y_ has been used). As with the non-magnetic polymers, the backbones of the MMIPs and MNIPs were similar, as were the microwave synthesised magnetic polymers.

**Figure 3:**
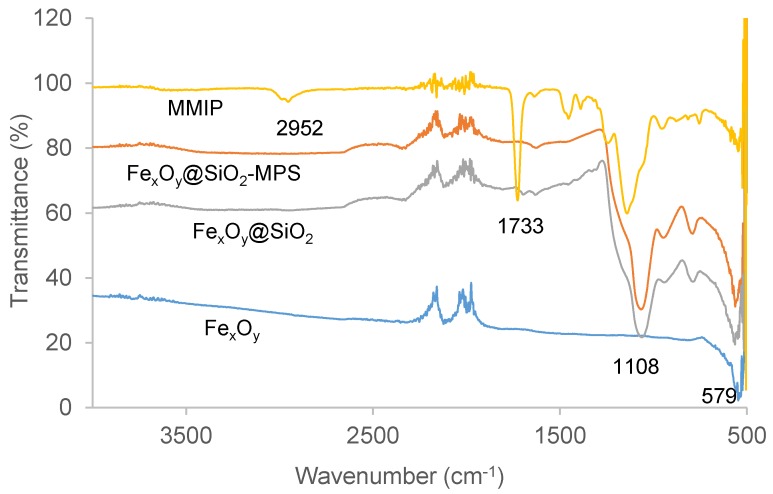
Overlaid Fourier transform-infrared (FTIR) spectra of Fe_x_O_y_ nanoparticles, Fe_x_O_y_@SiO_2_, Fe_x_O_y_@SiO_2_-MPS, and putative MMIP. Fe_x_O_y_@SiO_2_: Fe_x_O_y_ nanoparticles modified with SiO_2_; Fe_x_O_y_@SiO_2_-MPS: surface-modified magnetic particles; MPS: 3-(trimethoxysily)propyl methacrylate.

Attenuated total reflectance Fourier transform infrared (ATR-FTIR) analysis was performed to further ensure the correct preparation of polymers. Figure 3 shows the FTIR spectra of Fe_x_O_y_, Fe_x_O_y_@SiO_2_, Fe_x_O_y_@SiO_2_-MPS, and MMIP. In line with the results of Chen et al. [25], the adsorption peak at 579 cm^−1^ found in each spectra was indicative that Fe_x_O_y_ nanoparticles were present in these materials. The peaks around 1108 cm^−1^ were attributed to Si-O-Si, revealing the formation of the silica shell. The strong peak at 1733 cm^−1^ associated with the C=O functional group, and the lack of a peak at 1660 cm^−1^ ordinarily attributable to C=C, indicated the successful formation of MMIP by polymerisation of magnetic nanoparticle-bound 3-(trimethoxysily)propyl methacrylate (MPS), ethylene glycol dimethacrylate (EGDMA), and methyl methacrylate (MMA). The peak at 2952 cm^−1^, indicative of C–H stretches from methyl and methylene groups, confirmed MMIP coupling with MMA and EGDMA [36]. The FTIR spectra of MNIP, MW MMIP, and MW MNIP coincided with MMIP, and MIP had quite similar FTIR spectra (not shown), except without an adsorption peak of Fe–O at 579 cm^−1^. No variations were found between the different batches of polymers.

### 2.3. Adsorption Isotherms

**Figure 4:**
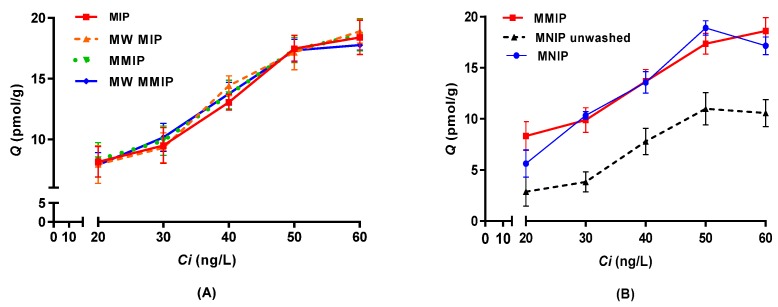
Adsorption isotherms of 3-isobutyl-2-methoxypyrazine (IBMP) (mean ± SD, standard deviation, *n* = 3) for (**A**) conventional and MW putative imprinted polymers and (**B**) thermally synthesised MMIP, MNIP, and unwashed MNIP. *Q*: equilibrium adsorption amount; *Ci*: initial IBMP concentration.

Binding tests were carried out to estimate the adsorption capability of polymers under different initial IBMP concentrations in model wine. The equilibrium isotherms for the adsorption of IBMP onto different imprinted polymers are shown in Figure 4A. The amount of IBMP binding to the polymers increased with increasing initial concentration and no difference was evident among the imprinted polymers according to one-way analysis of variance (ANOVA) and Tukey (HSD) pairwise comparison (*p* < 0.05), including those produced with a microwave or with the inclusion of iron oxide nanoparticles. Thus, under the low concentration range used in this study, the different forms of imprinted polymers could not be differentiated from each other.

The results of binding tests for thermally synthesised MMIP and MNIPs are shown in Figure 4B, where the adsorption amount also increased in line with the initial IBMP concentrations. The adsorption capability of imprinted polymers was not significantly different from the non-imprinted controls (one-way ANOVA, Tukey (HSD) pairwise comparison, *p* < 0.05). However, unwashed MNIP had a much lower binding capacity towards IBMP compared to MMIP, which could be due to cavities occupied by the trapped solvent. It was found that the washing process could not only remove template molecules, but also porogen solvent, from the polymers. This highlighted the importance of treating the non-imprinted polymers in exactly the same way as the imprinted polymers to provide proper controls [37].

Several linear and non-linear adsorption isotherm models were applied to fit the equilibrium data of thermally synthesised MMIP and MNIP. Linear models (Table 1) turned out to have a better fit based on their coefficients of determination (*R*^2^, Table 2 and Figure S1 of the Supplementary Materials). Polymers were consistently produced using the synthetic procedures outlined in Section 3.2 and batches produced identical results. Both Langmuir and Freundlich isotherms were a good fit for the experimental data. The Langmuir isotherm assumes monolayer adsorption with all sites equivalent to form a homogeneous surface. Once a molecule occupies a binding site, no further adsorption may take place at the same site and a saturation adsorption will be reached, also known as maximum adsorption. On the other hand, the Freundlich isotherm is used to describe surface heterogeneity assuming multilayer adsorption [38]. Though they are based on different theories, both Langmuir and Freundlich models might adequately describe the adsorption at certain concentrations, especially when the concentrations are low and the adsorption capacity of the adsorbent is large enough to make both isotherm equations approach linearity. In the present case, the analytical window is narrow and deliberately limited due to the practical concentration of IBMP in grapes and wines, compared to a concentration range that usually differs by at least two orders-of-magnitude for isotherm determinations. The resultant isotherm may ultimately correspond to only a subset of the sites in MMIP, and while informative, this could be inaccurate and inconsistent for estimating the binding properties in general [39]. Nonetheless, the *m* value of the Freundlich isotherm ranging from 0 to 1 indicates surface heterogeneity, where approaching zero means greater heterogeneity. The *m* value of 0.5436 for MMIP suggests that some heterogeneity was present; however, a more homogeneous surface could be assumed when the *m* value ranges between 0.5 and 0.9 [40]. The *m* value of 0.8822 suggested a more homogeneous surface for MNIP within the tested range, which is in line with the Langmuir assumption. Similar observations were also found in previous studies where the surface of imprinted polymers was more heterogeneous than that of the non-imprinted polymers [39]. In addition, the heterogeneity may also be caused by the addition of templates. *K* value (Dubinin-Radushkevich) relates to the free energy *E* (kJ/mol) of adsorption per molecule of adsorbate when it is transferred to the surface of the solid from infinity in the solution. *K* < 1 represents a rough surface with many cavities, and chemisorption can be assumed when the value of *E* is over 40 kJ/mol [40]. Thus, chemisorption could be expected for the polymers based on values of 1000 and 316 kJ/mol for MMIP and MNIP, respectively.

As shown in the isotherm graph (Figure 4B), specific binding was not observed in the adsorption analysis using model wine. This may be a result of the polymers being synthesised in a non-polar environment (toluene) rather than a wine matrix. A polar environment such as model wine (water, ethanol, tartaric acid) would destabilise the prearranged polymer complex [42] and MIPs should yield a better adsorption performance (relative to NIPs) in the same solvent as they were made [43,44].

### 2.4. Adsorption Dynamic

**Figure 5:**
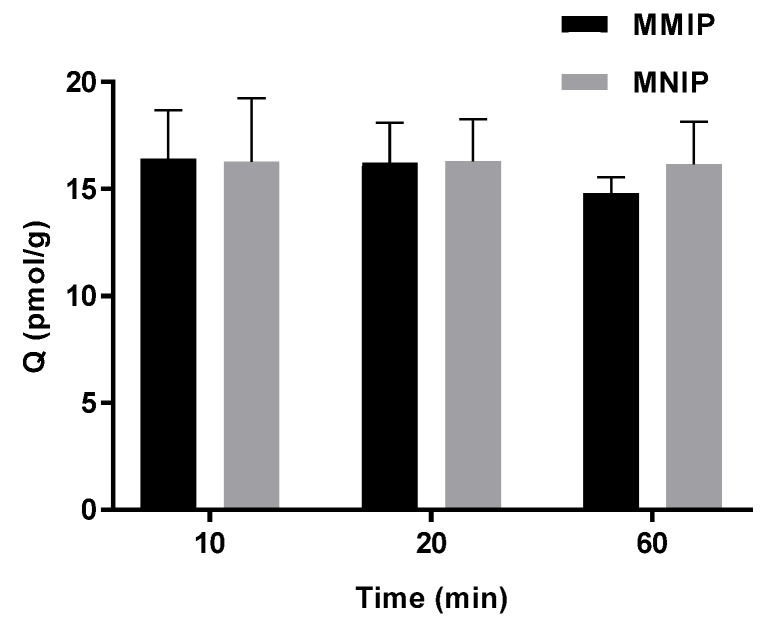
Kinetics for adsorption of 30 ng/L IBMP in model wine (mean ± SD *n* = 3) using thermally synthesised MMIP and MNIP.

Kinetic adsorption tests were carried out for thermally synthesised MMIP and MNIP in model wine spiked with 30 ng/L of IBMP. Adsorption equilibrium was reached within ten minutes for both polymers (Figure 5), which indicates prompt adsorption. No difference was found for time and polymer type according to one-way ANOVA with Tukey (HSD) pairwise comparison (*p* < 0.05).

### 2.5. Regeneration of Polymers.

**Figure 6:**
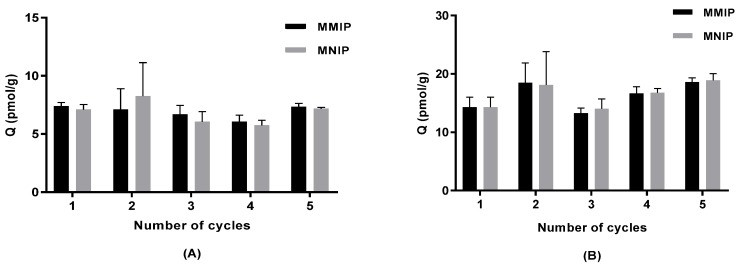
Adsorption on thermally synthesised magnetic polymers that were washed and cycled in model wine (mean ± SD, *n* = 3) for (**A**) 20 ng/L IBMP and (**B**) 50 ng/L IBMP.

Thermally synthesised magnetic polymers were washed (details in Section 3.4) and tested for reusability with low (20 ng/L) and high (50 ng/L) concentrations of IBMP spiked into model wine. MMIP and MNIP remained the same (one-way ANOVA, Tukey (HSD) pairwise comparison, *p* < 0.05) in terms of adsorption ability under both IBMP concentrations after cycling polymers five times (Figure 6).

### 2.6. Analysis of IBMP Adsorption in Spiked White Wine Samples

**Figure 7:**
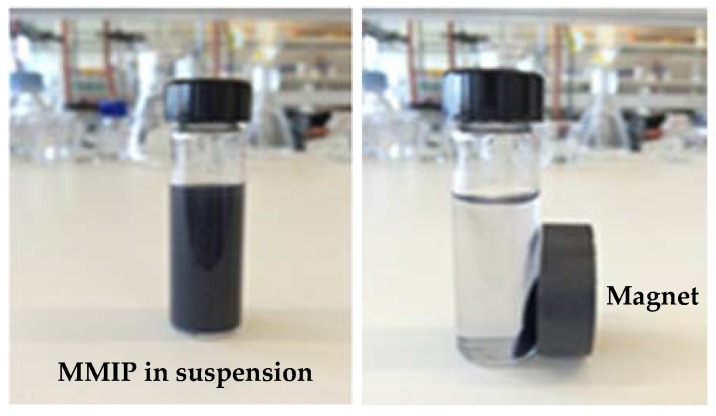
Magnetic separation of putative MMIP in white wine samples.

Two commercial Sauvignon Blanc wines, one from Australia (Aus, 0.4 ng/L IBMP) and the other from New Zealand (NZ, 14.1 ng/L), were spiked with 30 ng/L of IBMP (yielding IBMP concentrations prior to treatment as shown in Table 3) and used for adsorption testing. Magnetic separation was realised by placing a permanent magnet beside the vial containing the magnetic polymer (Figure 7). There was a lack of significant difference (one-way ANOVA, Tukey (HSD) pairwise comparison, *p* < 0.05) in the equilibrium adsorption amounts (*Q*) and percent adsorption of IBMP by MMIP and MNIP within the same wine. Interestingly, despite the wines being spiked with the same amount of IBMP, a higher amount of adsorption was observed for the NZ wine due to its higher initial IBMP concentration, which matches the adsorption isotherm data (Figure 4). The adsorption of IBMP on MMIP and MNIP in white wine was in line with that of adsorption in model wine solutions, including adsorption amount and binding properties. Overall, for a wine containing elevated IBMP levels (20 ng/L or above), an adsorption ability of up to 45% (using 1% *w*/*v* of polymer) and the reusability of the polymers described in Section 2.5 indicates they could decrease IBMP to a level below its sensory detection threshold (i.e., <10 ng/L) with perhaps single and certainly multiple treatments.

## 3. Materials and Methods

### 3.1. Chemicals

High purity solvents were purchased from Chem-Supply (Adelaide, SA, Australia). Iron (II, III) oxide particles (nanopowder, 50–100 nm particle size, 97% trace metals basis) and analytical reagent grade chemicals were purchased from Sigma-Aldrich (Castle Hill, NSW, Australia). d_3_-IBMP (99.9 atom% D) was supplied by C/D/N Isotopes Inc. (Point-Claire, QC, Canada). Water was obtained from a Milli-Q purification system (Millipore, North Ryde, NSW, Australia).

### 3.2. Preparation of Polymers

**Figure 8:**
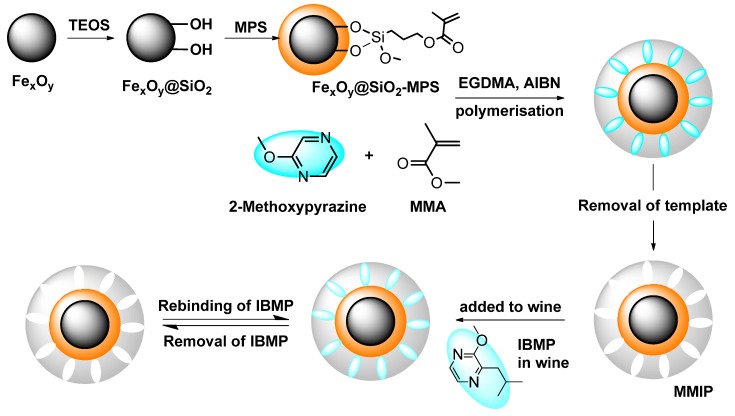
Schematic showing the preparation steps used in an attempt to produce MMIPs. TEOS: tetraethoxysilane; MMA: methyl methacrylate; EGDMA: ethylene glycol dimethacrylate; AIBN: 2, 2′-azobisissobutyronitrile.

Magnetic polymers were prepared by multi-step polymerisation, as shown in Figure 8. Fe_x_O_y_@SiO_2_-MPS nanoparticles were prepared first as magnetic bases using commercial iron (II, III) oxide particles instead of preparing through chemical co-precipitation. Surface modification of Fe_x_O_y_ followed the procedures of Chen et al. [25], with modification of the process of Zhang et al. [45], Zeng et al. [46], and Lu et al. [47]. The attempted MMIPs were then prepared according to Belbruno et al. [23] with some modifications. Briefly, MMA (432 μL, 4 mmol) was added to ‘dummy’ template molecule 2-methoxypyrazine (98 μL, 1 mmol) in toluene (12 mL) as a functional monomer. The obtained Fe_x_O_y_@SiO_2_-MPS nanoparticles (1 g) were then added and the mixture was stirred for 2 h at ambient temperature. After this time, cross-linker EGDMA (3.8 mL, 20 mmol) was added along with the initiator 2, 2′-azobisissobutyronitrile (AIBN, 100 mg). The mixture was degassed in an ultrasonic bath for 15 min and purged with nitrogen, sealed, and placed in a 60 °C oil bath for 24 h of polymerisation. The obtained bulk polymers were crushed and separated from the round-bottom flask. The polymers were dried under high vacuum and ground in a ball mill (full-directional planetary ball mill (QXQM-1), Tencan, Changsha, China). Ground polymer was passed through a 150 μm sieve (Retsch test sieve, 200 mm × 50 mm, 150 μm, VWR, Tingalpa, QLD, Australia) and the collected particles were washed with diethyl ether by Soxhlet extraction until no further 2-methoxypyrazine was detected in the washing solvent by gas chromatography-mass spectrometry (GC-MS) analysis [23]. MNIPs were made by the same protocol without addition of the 2-methoxypyrazine as a template. Other putative imprinted and non-imprinted polymers (i.e., MW MMIPs and MW MNIPs) were prepared in the same way as described above, except that the polymerisation process was completed within 1 h at 60 °C in a microwave synthesiser (CEM microwave synthesiser, Discover S, DKSH, Melbourne, VIC, Australia). Reactions were performed in a 35 mL sealed reaction vessel (CEM, DKSH). The microwave power was on in dynamic mode with a pressure limit of 150 psi.

A range of non-magnetic polymers (the MIPs, NIPs, MW MIPs, and MW NIPs) were also prepared in the same way, without the magnetic substrates. All the polymers were prepared in duplicate and gave similar yields of around 92%.

### 3.3. Characterisation of Polymers

IR spectra of ground polymers were measured by ATR on a PerkinElmer Spectrum 400 FTIR Spectrometer (Scientific Partners, Canning Vale, WA, Australia) in the 4000–500 cm^−1^ region. SEM images were obtained using an FEI Quanta 450 FEG environmental scanning electron microscope (ThermoFisher, Scoresby, VIC, Australia). The powdered samples were prepared by adhesion to carbon tabs and coated with platinum. SEM images were obtained at an accelerating voltage of 10 kV and magnification of 50,000× with a working distance of 10 mm. The images were taken under the same conditions.

### 3.4. Adsorption Equilibrium and Reusability of Polymers

Each polymer (10 mg/mL) was added to model wine solution (12 mL of 12% *v*/*v* EtOH and 5 g/L tartaric acid in MilliQ water, adjusted to pH 3.4 with 10 M NaOH) containing IBMP (99%, Sigma-Aldrich) at concentrations ranging from 20 ng/L to 60 ng/L. After shaking with an incubator at ambient temperature for 2 h at 120 rpm, polymers were separated by centrifugation (3857 rcf, 20 °C, 10 min) (Hettich, Universal 320/320R, Adelab, Adelaide, SA, Australia) and the supernatants (10 mL) were transferred into 20 mL headspace vials and measured by GC-MS as described below. All adsorption tests were conducted in triplicate.

The equilibrium adsorption amounts of IBMP (*Q* pmol/g) were calculated according to the following equation:

$$Q=\frac{\left(Ci-Cf\right)V}{WM}$$

where *Ci* and *Cf* (pg/mL) are the initial and final equilibrium concentrations of IBMP, respectively; *V* (mL) is the volume of IBMP model wine solution; *M* is the molar mass of IBMP; and *W* (g) is the amount of polymer added to the model wine solution. The unit of *Q* was adjusted in accordance with the unit of *C*, so pmol/g was used in accordance with ng/L, where µmol/g is in accordance with mg/L [25].

For kinetic adsorption tests, thermally synthesised MMIP and MNIP (10 mg/mL) were added into model wine solutions containing 30 ng/L of IBMP. After shaking at 120 rpm at different time intervals (10, 20, 60 min) at ambient temperature, the supernatants were separated by centrifugation and assessed by GC-MS. All adsorption tests were conducted in triplicate.

Thermally synthesised MMIP and MNIP were tested for reusability. Used polymers were immersed in diethyl ether and stirred for several hours to remove IBMP. The high vacuum dried polymers (10 mg/mL) were added to model wine with low (20 ng/L) and high (50 ng/L) IBMP concentrations. After shaking at ambient temperature for 1 h at 120 rpm, polymers were separated by centrifugation and the supernatants were analysed by GC-MS. All reuse tests were conducted in triplicate.

### 3.5. Adsorption of 3-Isobutyl-2-methoxypyrazine in Spiked White Wine Samples

Thermally synthesised MMIP and MNIP (10 mg/mL) were added separately into 10 mL of an Australian Sauvignon Blanc wine (12.5% *v*/*v* EtOH, Banrock Station, 2016) and a New Zealand Sauvignon Blanc wine (12.5% *v*/*v* EtOH, Wahu Marlborough, 2016), each spiked with 30 ng/L IBMP. The mixtures were shaken at ambient temperature for 30 min at 120 rpm. A permanent magnet was used to separate the polymers from solutions and supernatants (4 mL) were transferred into 20 mL headspace vials and diluted with 6 mL MilliQ water and adjusted to pH 6 with NaOH [48]. Further GC-MS analysis followed the procedure as detailed below. Selected ion monitoring (SIM) chromatograms are presented in Figure S2 of Supplementary Materials.

### 3.6. Headspace Solid-Phase Microextraction-Gas Chromatography-Mass Spectrometry Analysis of 3-Isobutyl-2-methoxypyrazine.

Samples were analysed with an Agilent 6890GC and 5973MSD equipped with a Gerstel MPS2 autosampler. Model wine (10 mL) or diluted wine samples (4 mL wine, 6 mL water) were added to 20 mL headspace vials, with 3 g NaCl and 10 µL of 50 µg/L d_3_-IBMP (in absolute ethanol) as the internal standard. Standard curves were created using model wine or diluted model wine (2.5-fold dilution) spiked with IBMP (solutions in absolute ethanol) ranging from 5–60 ng/L, in addition to 50 ng/L d_3_-IBMP. A 1 cm 23 gauge DVB/CAR/PDMS (Supelco, Sigma-Aldrich) solid-phase microextraction (SPME) fibre was used for undiluted model wine analysis and a 2 cm SPME fibre was used for white wine analysis. Sampling and instrumental analysis followed the method described by Chapman et al. [49].

### 3.7. Data Analysis

Significant differences between treatments were determined by one-way ANOVA with Tukey’s HSD multiple comparison test at *p* < 0.05 using XLSTAT (version 2014.5.03, Addinsoft, Paris, France). Graphs were processed using GraphPad Prism 7.02 (La Jolla, CA, USA).

## 4. Conclusions

Magnetic polymers were synthesised (including in a microwave), characterised, and found to remove up to 40% or more of the IBMP in model wine solutions and two white wines. Langmuir and Freundlich isotherm models were used to evaluate polymer binding properties within practical ranges of IBMP concentration in model wine. No difference in comparison to conventional synthesis was observed for the microwave polymers in the adsorption of IBMP from model wine and in physical characters by SEM and FTIR analysis. Furthermore, the addition of the magnetic substrate had no effect on the physical characters and binding properties of the polymers. The easy separation, reasonable adsorption ability towards IBMP, and regeneration ability make magnetic polymers an attractive potential option to remediate wines with elevated MP concentrations. However, the choice of polymer system needs further investigation to improve the specificity of the polymers. Binding of IBMP in model wine and white wine was apparently driven by non-specific hydrophobic interactions and the putatively synthesised molecularly imprinted polymer turned out to be no better at IBMP removal than its non-imprinted counterpart. Two components of the imprinting system could be examined further to improve the specificity. Firstly, the functional monomer could be considered. Compared to MMA, for instance, methacrylic acid has more active carboxylic acid functional groups to interact with the template and reinforce H-bonding [50]. Secondly, the solvent used to prepare the polymers could be optimised. Polymers were prepared using toluene, which acted as a porogen that has a similar size and structure to the target molecule. Thus, it may be that the template did not impart a distinctive enough shape to the polymers [51]. Studies could also include control imprinted polymers prepared with an unrelated template to verify in the event of greater binding that it was from molecular imprinting and not because of physical differences between the polymers [52]. Finally, for the practical usage of MMIPs, examination of the effects of polymers on other wine volatiles, colour parameters, and sensory properties also needs to be further studied.

## Figures and Tables

**Table 1:** List of linear form adsorption isotherm models.

Isotherm	Equation		Plot
Langmuir Type 2 [41]	$\frac{1}{Q}=\frac{1}{KQmax}\frac{1}{Cf}+\frac{1}{Qmax}$	*Q* (pmol/g): amount of IBMP adsorbed at equilibrium. *Cf* (ng/L): final equilibrium concentration of IBMP. *Q_max_* (pmol/g): maximum adsorption capacity. *K* (L/nmol): Langmuir adsorption equilibrium constant.	$\frac{1}{Q}\;Vs\;\frac{1}{Cf}$
Freundlich [25]	logQ=mlogCf+loga	*m*: adsorption intensity or surface heterogeneity. *a* (pmol/g): adsorption capacity of IBMP.	logQ Vs logCf
Dubinin-Radushkevich [25]	$lnQ=K{Ɛ}^{2}+lnQmax$ $Ɛ=RTln\left(1+\frac{1}{Cf}\right)$ $E={\left(-2K\right)}^{-1/2}$	*K* (kJ^2^/mol^2^): Dubinin-Radushkevich constant. *Ɛ*: Polanyi potential. *E* (kJ/mol): mean adsorption energy. *R*: gas constant (8.314 J/mol/K). *T* (*K*): absolute temperature.	$lnQ\;Vs\;{Ɛ}^{2}$

**Table 2:** Langmuir, Freundlich, and Dubinin-Radushkevich isotherm constants for the adsorption of IBMP on thermally synthesised magnetic polymers.

	MMIP	MNIP
***Langmuir Type 2***		
*Q_max_* (pmol/g)	25.19	84.03
*K* (L/nmol)	0.3028	0.016
***R*** ^2^	0.9688	0.8842
Freundlich		
*a* (pmol/g)	6.24	1.99
*m*	0.5436	0.8822
***R*** ^2^	0.9598	0.7754
***Dubinin-Radushkevich***		
*Q_max_* (pmol/g)	16.95	21.60
*K* (kJ^2^/mol^2^)	5 × 10^−7^	−5 × 10^−6^
*E* (kJ/mol)	1000	316
***R*** ^2^	0.8710	0.9556

**Table 3:** Adsorption of IBMP (mean ± SD, *n* = 3) by thermally synthesised MMIP and MNIP in commercial Sauvignon Blanc wines spiked with IBMP.

	Australia (Aus)	New Zealand (NZ)
Original IBMP (ng/L)	0.4 ± 0.1	14.1± 0.4
Spiked with 30 ng/L IBMP	25 ± 3	38 ± 2
*Q*_MMIP_ (pmol/g) *Q*_MNIP_ (pmol/g)	6 ± 3 3 ± 2	10 ± 3 9 ± 2
MMIP adsorption (%) MNIP adsorption (%)	42 ± 19 19 ± 10	45 ± 11 38 ± 5

## Supplementary Materials

The following are available online, Figure S1: Linear isotherm analysis plots of thermally synthesised magnetic polymers showing (a) Langmuir Type 2 analysis plot of putative magnetic molecularly imprinted polymer (MMIP), (b) Langmuir Type 2 analysis plot of magnetic non-imprinted polymer (MNIP), (c) Freundlich analysis plot of putative MMIP, (d) Freundlich analysis plot of MNIP, (e) Dubinin-Radushkevich analysis plot of putative MMIP, and (f) Dubinin-Radushkevich analysis plot of MNIP; Figure S2: Gas chromatography-mass spectrometry selected ion monitoring chromatograms of white wines showing (a) spiked Australian Sauvignon Blanc, (b) spiked Australian Sauvignon Blanc after putative MMIP treatment, (c) spiked New Zealand Sauvignon Blanc, and (d) spiked New Zealand Sauvignon Blanc after putative MMIP treatment.
