# Supplementary material for: Preparation of Magnetic Polymers for the Elimination of 3-Isobutyl-2-methoxypyrazine from Wine
Source: Molecules. 2018 May 10;23(5):1140. doi: 10.3390/molecules23051140 (PMC6099604; doi:10.3390/molecules23051140)
Supplement: Supplementary file 1 [file molecules-23-01140-s001.pdf]

Supplementary Information for

Preparation of magnetic polymers for elimination of 3-isobutyl-2-methoxypyrazine from wine

Chen Liang, David W. Jeffery* and Dennis K. Taylor

ARC Training Centre for Innovative Wine Production and School of Agriculture, Food and Wine, The University of Adelaide, Waite Campus, PMB 1, Glen Osmond, SA 5064, Australia; c.liang@adelaide.edu.au (C.L.); dennis.taylor@adelaide.edu.au (D.K.T.)

*Correspondence: david.jeffery@adelaide.edu.au; Tel.: +61 8 8313 6649

Table of Contents

Page

Figure S1. Linear isotherm analysis plots of thermally synthesised magnetic polymers S-2

Figure S2. Gas chromatography-mass spectrometry selected ion monitoring chromatograms of white wines S-3

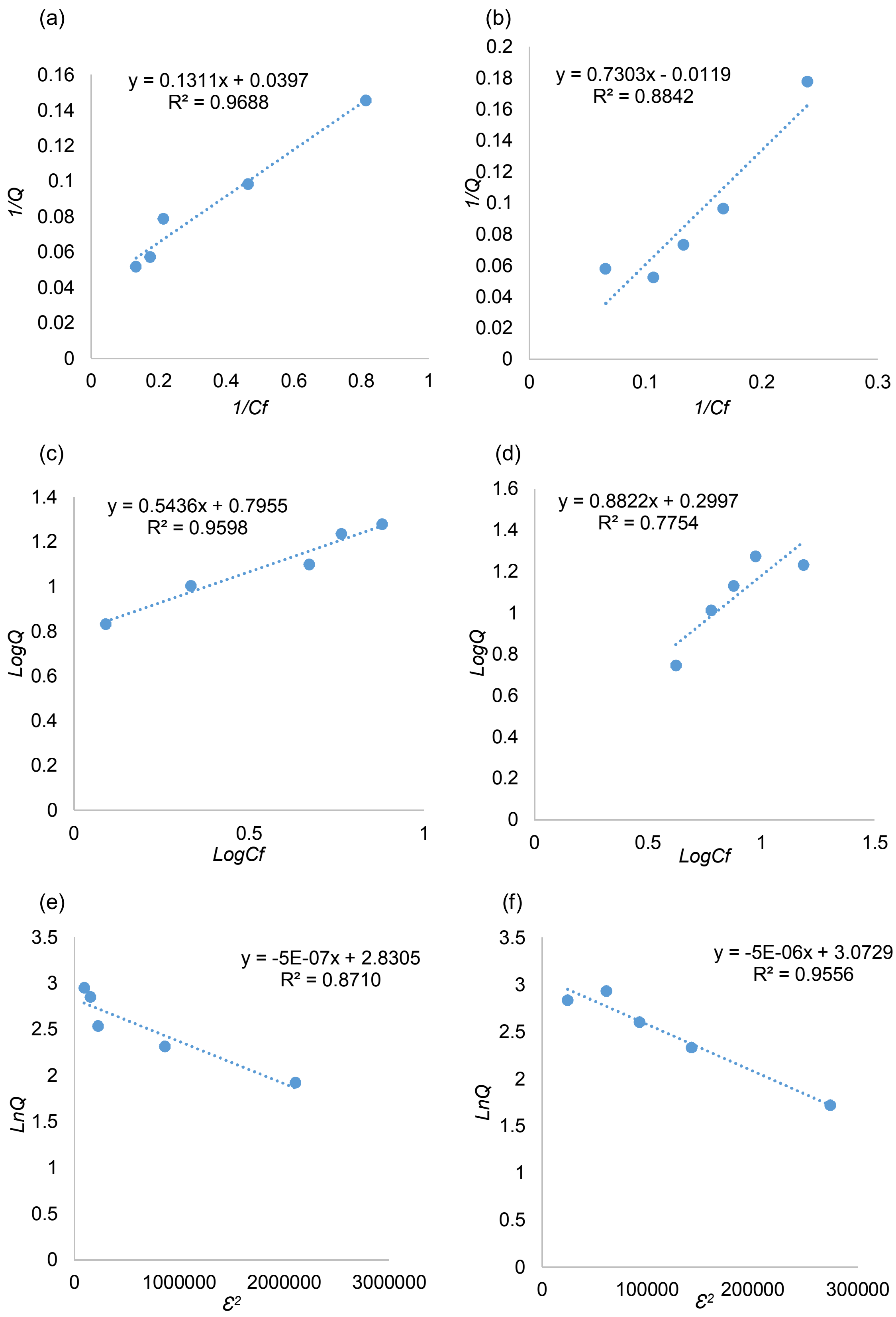

Figure S1. Linear isotherm analysis plots of thermally synthesised magnetic polymers showing (a) Langmuir Type 2 analysis plot of putative magnetic molecularly imprinted polymer (MMIP), (b) Langmuir Type 2 analysis plot of magnetic non-imprinted polymer (MNIP), (c) Freundlich analysis plot of putative MMIP, (d) Freundlich analysis plot of MNIP, (e) Dubinin-Radushkevich analysis plot of putative MMIP, and (f) Dubinin-Radushkevich analysis plot of MNIP. *Q*: equilibrium adsorption amount (pmol/g); *Cf*: final equilibrium concentration of 3-isobutyl-2-methoxypyrazine (IBMP) (ng/L); *Ɛ*: polanyi potential.

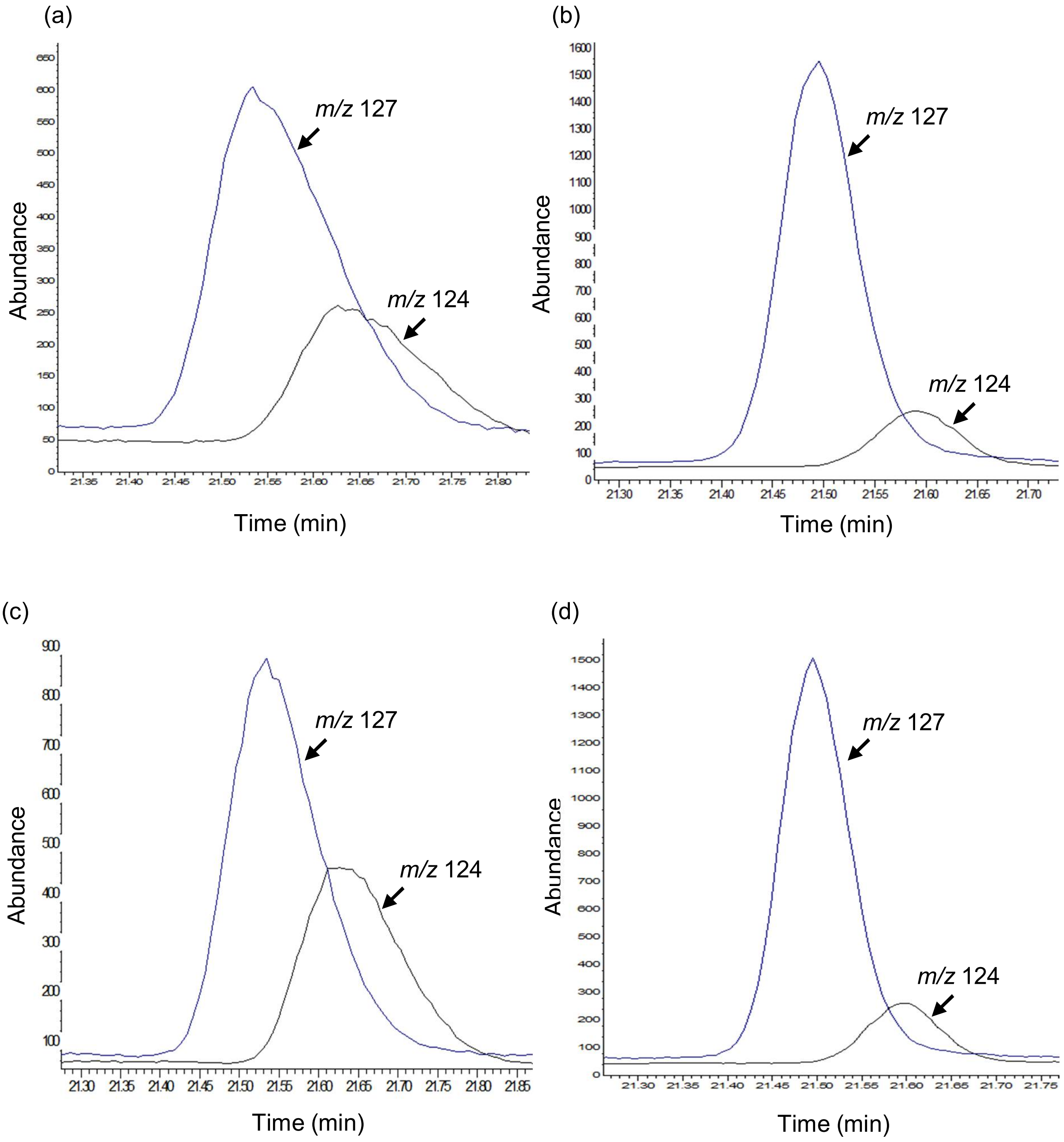

Figure S2. Gas chromatography-mass spectrometry selected ion monitoring chromatograms of white wines showing (a) spiked Australian Sauvignon Blanc, (b) spiked Australian Sauvignon Blanc after putative MMIP treatment, (c) spiked New Zealand Sauvignon Blanc, and (d) spiked New Zealand Sauvignon Blanc after putative MMIP treatment. IBMP was quantified using m/z = 124 with d_3-IBMP at m/z = 127 as the labelled internal standard.
